# The *Toxoplasma gondii* Active Serine Hydrolase 4 Regulates Parasite Division and Intravacuolar Parasite Architecture

**DOI:** 10.1128/mSphere.00393-18

**Published:** 2018-09-19

**Authors:** Ian T. Foe, Ouma Onguka, Katherine Amberg-Johnson, Rikki M. Garner, Neri Amara, Wandy Beatty, Ellen Yeh, Matthew Bogyo

**Affiliations:** a Department of Pathology, Stanford University School of Medicine, Stanford, California, USA; b Microbiology and Immunology, Stanford University School of Medicine, Stanford, California, USA; c Biophysics Program, Stanford University School of Medicine, Stanford, California, USA; d Department of Molecular Microbiology, Washington University School of Medicine, Saint Louis, Missouri, USA; e Department of Biochemistry, Stanford University School of Medicine, Stanford, California, USA; f Chan Zuckerburg Biohub, San Francisco, California, USA; University at Buffalo

**Keywords:** ASH proteins, serine hydrolase, cell division, intravacuolar organization

## Abstract

Hydrolase are enzymes that regulate diverse biological processes, including posttranslational protein modifications. Recent work identified four active serine hydrolases (ASHs) in Toxoplasma gondii as candidate depalmitoylases. However, only *TgPPT1* (*ASH1*) has been confirmed to remove palmitate from proteins. *ASH4* (TgME49_264290) was reported to be refractory to genetic disruption. We demonstrate that recombinant ASH4 is an esterase that processes short acyl esters but not palmitoyl thioesters. Genetic disruption of *ASH4* causes defects in cell division and premature scission of parasites from residual bodies. These defects lead to the presence of vacuoles with a disordered intravacuolar architecture, with parasites arranged in pairs around multiple residual bodies. Importantly, we found that the deletion of *ASH4* correlates with a defect in radial dispersion from host cells after egress. This defect in dispersion of parasites is a general phenomenon that is observed for disordered vacuoles that occur at low frequency in wild-type parasites, suggesting a possible general link between intravacuolar organization and dispersion after egress.

**IMPORTANCE** This work defines the function of an enzyme in the obligate intracellular parasite Toxoplasma gondii. We show that this previously uncharacterized enzyme is critical for aspects of cellular division by the parasite and that loss of this enzyme leads to parasites with cell division defects and which also are disorganized inside their vacuoles. This leads to defects in the ability of the parasite to disseminate from the site of an infection and may have a significant impact on the parasite's overall infectivity of a host organism.

## Abstract (precis)

This work defines the function of an enzyme in the obligate intracellular parasite Toxoplasma gondii. We show that this previously uncharacterized enzyme is critical for aspects of cellular division by the parasite and that loss of this enzyme leads to parasites with cell division defects and which also are disorganized inside their vacuoles. This leads to defects in the ability of the parasite to disseminate from the site of an infection and may have a significant impact on the parasite's overall infectivity of a host organism.

## INTRODUCTION

Toxoplasma gondii is an obligate intracellular pathogen from the parasitic phylum Apicomplexa, which contains many important human and agricultural pathogens such as Plasmodium falciparum, Cryptosporidium parvum, and Babesia microti. T. gondii is estimated to infect nearly 30% of the world’s population (1). Infection by T. gondii is generally tolerated, except when the host is immunocompromised or pregnant (2). In the immunocompromised subject, infection causes toxoplasmosis, symptoms of which include blindness, seizures, and, in some cases, death (1, 2). Women infected during pregnancy can pass the parasite to the neonate, causing congenital toxoplasmosis resulting in blindness, mental retardation, and stillbirth (2).

The ability of T. gondii to cause acute disease is dependent on its ability to complete its replication cycle (3). This cycle begins with the invasion of a host cell (4), which creates a protective vacuole around the parasite (5). The parasite asexually divides within the vacuole through a process called endodyogeny (6). During this process, daughter parasites form side by side in the cytosol of the mother (7). After formation of the daughters, the mother parasite undergoes cytokinesis from the apical end to the basal end of the parasite (7). This cytokinetic event is frequently incomplete and leaves the daughter parasites attached at their basal ends to a structure called the residual body (6–10). As a result, parasites are arranged around the residual body such that they resemble the petals of a flower (rosettes) (6, 8). After cell division, the parasites egress from the host cell (11), destroying it, and restart the replication cycle by infecting nearby cells.

One class of enzymes that is important for the successful completion of replication is the serine hydrolases (12, 13). Serine hydrolases are enzymes that utilize an activated serine residue and a water molecule to hydrolyze amide, ester, and thiol ester linkages in substrates (14). These enzymes have diverse substrates, including proteins and small molecules such as lipids and other ester-containing metabolites (14). Recent work has suggested that several members of the T. gondii active serine hydrolase (ASH) family are important for parasite replication (12, 13). In particular, chemical inhibition of the founding member of the ASH family (TgPPT1/ASH1) has confirmed its involvement in host cell invasion, parasite motility, and the secretion of adhesion/invasion organelles called micronemes (12). TgPPT1 functions as a depalmitoylase, cleaving thioester-linked palmitate groups from proteins such as TgGAP45, TgCDPK3, and TgARO (12). Hidden Markov modeling of the other family members prompted the suggestion that they may also function as depalmitoylases (13), although this has never been definitively demonstrated. Genetic knockouts of the ASH genes have provided little information on their cellular functions, as parasites lacking either *TgPPT1* or *ASH2-3* have no obvious phenotypes (12, 13, 15). Furthermore, *ASH4* (TgME49_264290) was previously reported to be “refractory to genetic deletion” (13), suggesting that it is likely critical for parasite growth.

Due to the suggested importance of *ASH4* in parasite biology (13), we sought to define its cellular function. Here we show that ASH4 likely functions as an esterase that cleaves short acyl esters and not as a depalmitoylase as previously suggested (13). Importantly, our results also suggest that ASH4 plays an unexpected role(s) in endodyogeny, intravacuolar parasite architecture, and the ability of parasites to disperse from the host cells after egress.

## RESULTS

### ASH4 functions *in vitro* as a short-acyl-chain esterase.

**FIG 1:**
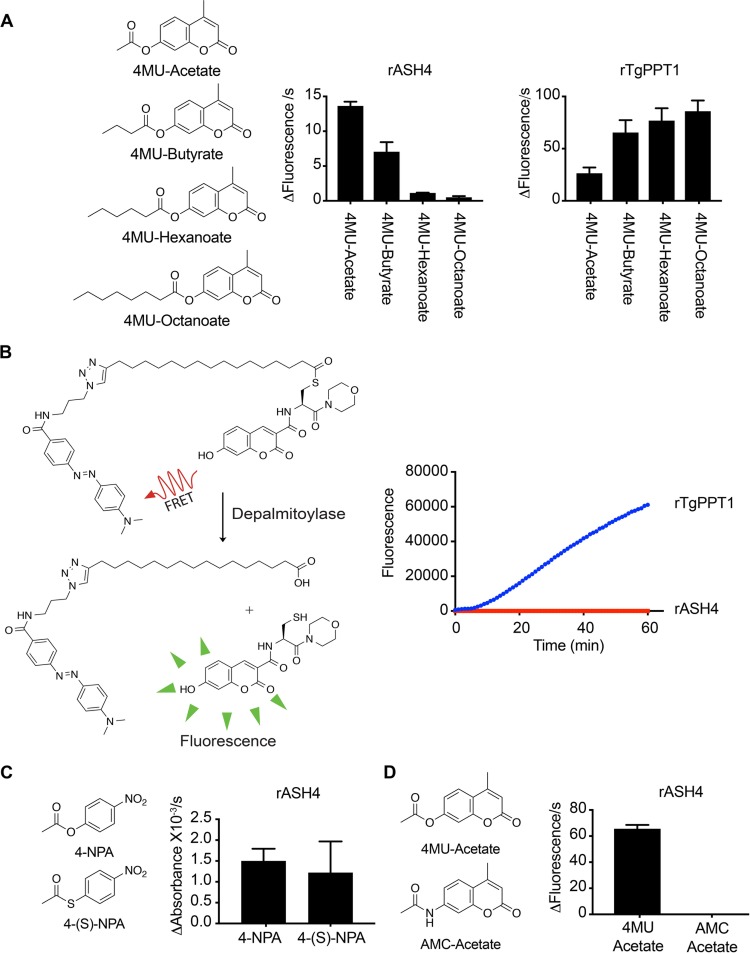
ASH4 is an acetyl esterase. (A) Structures of the 4-methyllumbelliferone ester substrates and their rates of processing by rASH4 and rTgPPT1. Graphs show the rate of fluorescence change per second, and data are plotted as averages of results from three independent experiments repeated in technical triplicate. (B) Structure of the quenched fluorogenic substrate, QStE, for depalmitoylase activity. The graph at the right depicts a representative progress curve for processing of the QStE substrate by rTgPPT1 and rASH4. Data represent averages of results from 3 technical replicates. Error bars show the standard deviations. (C) Structures of 4-nitrophenyl acetate (4-NPA; ester substrate) and 4-nitrothiolphenyl acetate (4-S-NPA; thioester substrate) substrates. The graph at right shows the average rate at which rASH4 cleaves each substrate. The graph depicts averages of results from three independent experiments repeated in technical triplicate. Error bars indicate standard errors of the means. (D) Structures of 4-methyl-2-oxo-2*H*-chromen-7-yl acetate (4MU-acetate; ester substrate) and *N*-(4-methyl-2-oxo-2*H*-chromen-7-yl) acetamide (AMC-acetate; amide substrate). The graph at right shows the average rate of hydrolysis of each substrate by rASH4. The graph represents results from three independent experiments repeated in technical triplicate. Error bars indicate standard errors of the means.

Homology modeling has suggested that *ASH4* is a member of a family of serine hydrolases with putative depalmitoylase activity (13). Depalmitoylase activity has been confirmed only for the founding member of the family, TgPPT1 (12, 13), and not for other family members (ASH2-4). To determine the substrate specificity of ASH4, we expressed and purified 6×His-tagged recombinant ASH4 (rASH4) and a control catalytically dead version in which we mutated serine 124 (putative active-site serine) to an alanine (rASH^S124A^) from Escherichia coli (see Fig. S1A in the supplemental material). To identify possible substrates, we examined the ability of rASH4 to hydrolyze a small library of fluorescent acyl ester substrates based on the 4-methyllumbelliferone fluorophore. The substrates in this library differ only by the length of the acyl chain (Fig. 1A). Unlike rTgPPT1, which prefers long-chain lipid esters, we found that rASH4 preferred short acyl ester substrates, with the two-carbon acetyl ester having the highest rate of hydrolysis (Fig. 1A). Importantly, hydrolysis was dependent on the active-site serine, as rASH4^S124A^ had no activity in these assays (Fig. S1B). While the preference for short acyl chains suggests that rASH4 is not a depalmitoylase, we sought to directly test its ability to cleave palmitoyl thioesters using a substrate (QStE) recently developed in our group that measures the thioesterase activity of depalmitoylases. The QStE substrate contains an S-palmitoylated cysteine residue with a fluorophore on its N terminus and a fluorophore quencher on its palmitoyl group. When the palmitate quencher is removed by hydrolysis, fluorescence increases (Fig. 1B). Consistent with ASH4 failing to cleave long-chain lipid esters, we found that, unlike TgPPT1, rASH4 was completely unable to process the QStE substrate (Fig. 1B). These data strongly suggest that while ASH4 is similar (32% identity) to TgPPT1 at the amino acid level, it is unlikely to be a depalmitoylase.

10.1128/mSphere.00393-18.1 FIG S1 Recombinant expression and activity of ASH4. (A) Coomassie-stained gel image (left) showing expression and purification of recombinant ASH4 (rASH4). A Coomassie-stained gel (right) shows expression and purification of recombinant catalytically dead ASH4 (rASH4^S124A^). NI, not-induced sample; I, induction sample; FT, flowthrough; BM, bead-bound material; E, elution from beads. The arrow indicates rASH4 and rASH4^S124A^. (B) Structures of ester substrates and graph of a representative progress curve for rASH4^S124A^ cleavage of the substrates. Error bars show data ± standard deviations (SD). Results of cleavage of 4MU-acetate by rASH4 are shown as a positive control. The graph represents average results from 3 technical replicates. (C) Substrate structures and graph of a representative progress curve for rASH4 cleavage of 4-methyl-2-oxo-2*H*-chromen-7-yl acetate (4MU-Acetate) and *N*-(4-methyl-2-oxo-2*H*-chromen-7-yl) acetamide (AMC-Acetate) ± SD. The graph represents averages of results from three technical replicates. Download FIG S1, PDF file, 0.2 MB. Copyright © 2018 Foe et al. 2018 Foe et al. This content is distributed under the terms of the Creative Commons Attribution 4.0 International license.

Acetyl groups can be conjugated to proteins and other small molecules via a number of chemical bonds such as amides, esters, and thioesters. Serine hydrolases have been reported to cleave all three of these bond types (14). We therefore tested the ability of rASH4 to cleave an acetyl ester, a thioester, or an amide substrate. We found that rASH4 efficiently processed both acetyl esters and thioesters but not the acyl amide substrate (Acetyl-AMC) (Fig. 1C and D; see also Fig. S1C and D). These results suggest that ASH4 primarily cleaves short-acyl-chain esters and thioesters and is unlikely to be a deacylase of amides such as acyl lysine residues.

### ASH4 is important for the ordered intravacuolar architecture of parasites.

**FIG 2:**
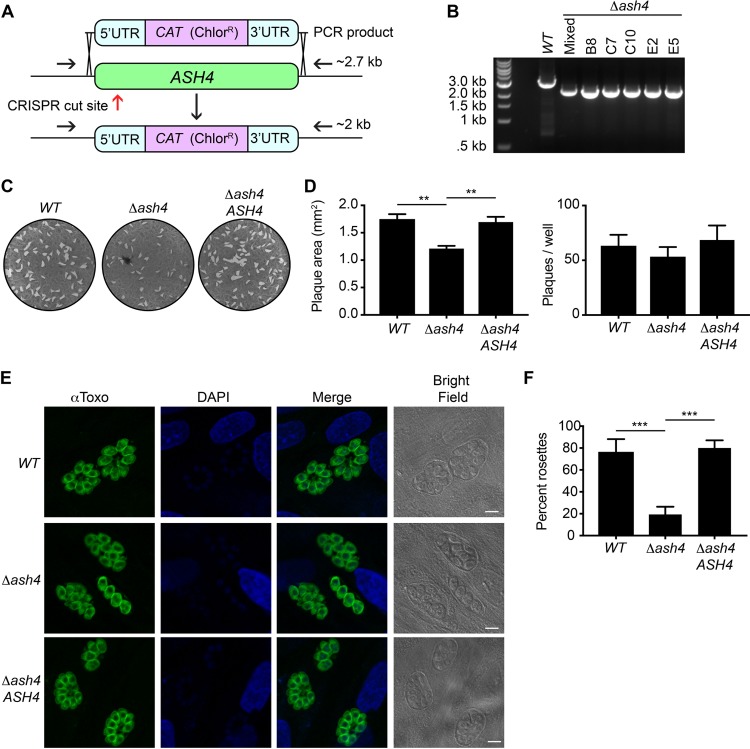
Loss of ASH4 results in disordered vacuoles and reduced area of infection *in vitro*. (A) Diagram of the *ASH4* knockout strategy. The red arrow indicates the approximate location of the CRISPR/Cas9 cut site. Black arrows indicate primers used to confirm *ash4* deletion. The intact ASH4 locus produces a band of ∼2.7 kb, while the *ash4* deletion results in a 2-kb band upon PCR amplification using the shown primer locations. (B) PCR results from the wild type (WT), a chloramphenicol-resistant population (Mixed), and five *Δash4* clones. Clone E2 was used in subsequent experiments. (C) Representative images of plaque assays from wild-type (*WT*), *Δash4*, and ASH4 rescue (*Δash4 ASH4*) parasites. (D) Quantification of average plaque size in square millimeters (left) and total plaque numbers (right). Data plotted are from 5 independent experiments performed in technical duplicate. Error bars indicate standard errors of the means. One-way analysis of variance (ANOVA) was performed with a subsequent Tukey’s multiple-comparison test to determine significance. **, *P* < 0.01. (E) Indirect immunofluorescence images of wild-type (*WT*), *Δash4*, and ASH4 rescue (*Δash4 ASH4*) parasites stained with anti-Toxo (α-Toxo) antibody and DAPI. Bright-field images are shown to highlight vacuoles. Scale bars are 5 μm. (F) Quantification of percentages of parasites in ordered rosettes for wild-type (*WT*), *Δash4*, and ASH4 rescue (*Δash4 ASH4*) parasites. The graph presents averages of results from three independent experiments in which at least 50 vacuoles were counted per strain. Error bars indicate the standard deviations. One-way ANOVA was performed with Tukey’s multiple-comparison test to determine significance, with all statistically significant comparisons indicated. ***, *P* < 0.001.

Previous reports have suggested that the *ASH4* gene is refractory to genetic disruption, prompting the suggestion that it is essential for growth of T. gondii (13). However, a genome-wide CRISPR/Cas9 screen demonstrated that the loss of *ASH4* had only a small effect on fitness *in vitro* (fitness score of −1.2), suggesting that it is likely not essential (15). We therefore used the CRISPR/Cas9 genome editing method (16) in an RH, *Δku80* strain to generate *Δash4* parasites (Fig. 2A), which were confirmed by PCR (Fig. 2B). The previous inability to generate *Δash4* parasites, along with the modest decrease in fitness associated with loss of *ASH4*, suggests that, while not essential, *ASH4* may play an important role in parasite replication.

As an initial attempt to identify specific phenotypes in the *Δash4* parasites, we performed plaque assays to monitor invasion, replication, egress, and overall infectivity of the parasites. We found that the wild-type and knockout parasites formed similar numbers of plaques (Fig. 2C and D). Interestingly, however, the *Δash4* parasites formed plaques that were significantly (∼30%) smaller than those seen with the wild type (Fig. 2C and D). This reduced plaque size was rescued by the introduction of wild-type *ASH4* at an exogenous site in the mutant (*Δash4 ASH4*) parasites (Fig. 2C and D; see also Fig. S2). These data strongly suggest that *ASH4*, while not essential, plays a role in parasite growth *in vitro*.

10.1128/mSphere.00393-18.2 FIG S2 Generation of the *ASH4* (*Δash4 ASH4*) rescue strain. The cartoon illustrates *ASH4* integration at the UPRT locus. Black arrows indicate primers for confirming the presence of ASH4. Primers lie both in the UTRs of the rescue construct and in the endogenous ASH4 locus. Primers were used to test for integration of the rescue construct and the *Δash4* status. Blue arrows indicate locations of UPRT integration check primers. The left gel shows PCR data confirming integration of the ASH4 rescue construct. PCR on ASH4 rescue strains generates both a 2-kb band indicating *Δash4* and a 1.3-kb band corresponding to the rescue construct. Two clones are shown for ASH4 rescue; clone C4 was used for the experiments described in the paper. The gel at the right shows successful integration of the rescue construct at the UPRT locus. Successfully integration results in a PCR product of 2.2 kb. Download FIG S2, PDF file, 0.4 MB. Copyright © 2018 Foe et al. 2018 Foe et al. This content is distributed under the terms of the Creative Commons Attribution 4.0 International license.

As T. gondii parasites divide, they frequently form vacuoles during cell division that have a unique intravacuolar architecture (6). The dividing parasites are arranged as rosettes, with the parasites organizing into a flower-shaped pattern centered on a structure called the residual body (6, 8). We observed that the *Δash4* parasites frequently failed to form rosettes and instead formed vacuoles with disordered patterns (Fig. 2E). This was most obvious after three rounds of division, where we found that nearly 80% of the wild-type parasites were able to form rosettes compared with only 20% of the *Δash4* parasites (Fig. 2F). The failure to form rosettes could be rescued by exogenous expression of the *ASH4* gene (*Δash4 ASH4*; Fig. 2E and F).

**FIG 3:**
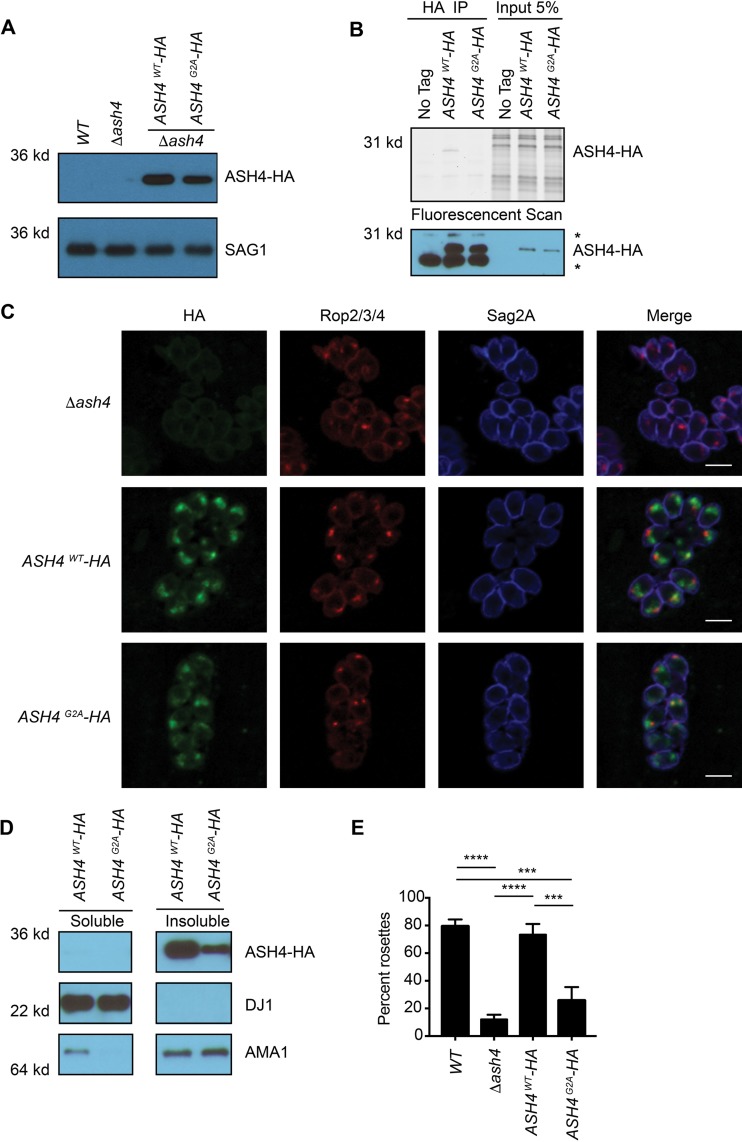
Myristoylation of ASH4 is important for ordered intravacuolar architecture. (A) Anti-HA Western blotting of WT, *ASH4* deletion, and ASH4^WT^-HA and ASH4^G2A^-HA strains showing protein expression levels. A SAG1 Western blot is shown as a loading control. (B) Parasites were metabolically labeled with a CLICKable myristic acid analog and subsequently lysed, and myristoylated proteins were labeled with rhodamine azide dye and immunoprecipitated with an anti-HA antibody (HA IP) or loaded directly (Input 5%). The upper gel image represents a fluorescent rhodamine scan for the WT, ASH4^WT^-HA, and ASH4^G2A^-HA lanes. The position of the labeled ASH4 is indicated (ASH4-HA). The anti-HA Western blot (lower panel) depicts levels of ASH4-HA present in each sample, *, nonspecific bands. (C) Indirect immunofluorescence microscopy of parasites expressing ASH4^WT^-HA and ASH4^G2A^-HA. Parasites were stained with anti-HA to show ASH4 localization, anti-ROP2/3/4 for rhoptories/apical end of parasites, and anti-SAG2A for the plasma membrane of parasites. The scale bar indicates 5 μm. (D) Anti-HA Western blot showing levels of ASH4^WT^-HA and ASH4^G2A^-HA protein in soluble and membrane lysate fractions. A SAG1 Western blot is shown to indicate the insoluble fraction. A DJ1 Western blot is shown to indicate the soluble fraction. The soluble and insoluble fractions are from the same gel and had the same exposure. (E) Graph depicting average percentages of parasites that formed ordered rosettes for wild-type (*WT*), *Δash4*, *ASH4^WT^-HA*, and *ASH4^G2A^-HA* parasites. The experiment was repeated three times with at least 50 vacuoles for each strain counted per experiment. Only vacuoles containing 8 parasites were counted. Error bars indicate standard deviations. One-way ANOVA was performed followed by Tukey’s multiple-comparison test to determine significance, with all statistically significant comparisons indicated. ***, *P* < 0.001; ****, *P* < 0.0001.

Proteins that have a glycine as their second amino acid are often posttranslationally modified with myristate, a 14-carbon fatty acid, which is frequently involved in protein localization and/or protein/protein interactions (17). To determine if ASH4 (which has a glycine in position 2) was myristoylated, we generated *Δash4* parasites that expressed either wild-type C-terminally HA tagged ASH4 (*ASH4^WT^-HA*) or ASH4 with the glycine-to-alanine mutation at position 2 (*ASH4^G2A^-HA*) (Fig. 3A; see also Fig. S3A). We then used a metabolic labeling strategy to determine if ASH4 is myristoylated. We incubated knockout and ASH4^WT^-HA- and ASH^G2A^-HA-expressing parasites overnight with myristate containing a terminal alkyne, which is a substrate for the endogenous N-myristoyl transferase (18). We then lysed parasites, immunoprecipitated ASH4 using the HA epitope, and performed an on-bead, copper (I)-catalyzed azide-alkyne cycloaddition “click” reaction with the azido-tetramethylrhodamine fluorophore. Analysis of the labeled samples by SDS-PAGE confirmed the presence of fluorescently labeled ASH4 only in the *ASH4^WT^-HA* strain, confirming that it was myristoylated at the G2 residue (Fig. 3B). To determine if myristoylation of ASH4 regulates its localization, we performed immunofluorescence microscopy with tyramide amplification using an anti-HA antibody in ASH4^WT^-HA- and ASH4^G2A^-HA-expressing parasites. Consistent with previous reports, ASH4^WT^-HA was localized to the apical end of the parasite. Surprisingly, the myristoylation-deficient ASH4^G2A^-HA strain still formed punctate apical foci (Fig. 3C), demonstrating that myristoylation is not essential for proper ASH4 localization.

10.1128/mSphere.00393-18.3 FIG S3 Generation of *ASH4^*WT*^-HA* and *ASH4^*G2A*^-HA* rescue strains. (A) Cartoon illustrating how the *ASH4-HA* alleles were introduced into the UPRT locus using the strategy described in the Fig. S2 legend. The left gel shows PCR data obtained using primers to demonstrate integration of ASH4-HA rescue construct. The right gel shows PCR with primers used for confirming construct integration into the UPRT locus. (B) The graph depicts average plaque sizes of wild-type (WT), *Δash4*, *ASH4^*WT*^-HA*, and *ASH4^*G2A*^-HA* parasites in square millimeters ± SEM. The experiment was performed 3 independent times in technical triplicate. One-way ANOVA was performed followed by Tukey’s multiple-comparison test to determine statistical significance; all statistically significant comparisons are indicated. *, *P* < 0.05. Download FIG S3, PDF file, 0.2 MB. Copyright © 2018 Foe et al. 2018 Foe et al. This content is distributed under the terms of the Creative Commons Attribution 4.0 International license.

Myristoylation can regulate protein localization by promoting transient association with lipid membranes (17). To determine if ASH4 was membrane associated, we used high-speed centrifugation to fractionate ASH4-HA lysates into soluble and insoluble fractions. Consistent with ASH4 being membrane associated, we found that the majority of ASH4^WT^-HA protein was in the insoluble fraction (Fig. 3D). Surprisingly, membrane association was not affected by myristoylation (Fig. 3D), suggesting that myristoylation is not required for proper ASH4 localization or its membrane association.

We next assessed the effect of ASH4 myristoylation on the formation of ordered rosettes. Parasites expressing the ASH4^WT^-HA protein formed rosettes at the same frequency as wild-type parasites, while parasites expressing the myristoylation-deficient ASH4^G2A^-HA strain produced predominantly disordered vacuoles, on par with what was observed in the knockout strain (Fig. 3E). Taken together, these data suggest that myristoylation, while not necessary for localization or association with the membrane, is important for the organization of parasites in the vacuole. To determine if ASH4 myristoylation was also important for parasite growth, we performed plaque assays with the ASH4^WT^-HA and ASH4^G2A^-HA parasites. Expression of either ASH4-HA or ASH4^G2A^-HA proteins failed to rescue the small-plaque phenotype (Fig. S3B), making it difficult to assess the contribution of myristoylation to the small-plaque phenotype in these strains.

### *Δash4* parasites are organized in the vacuole in pairs.

**FIG 4:**
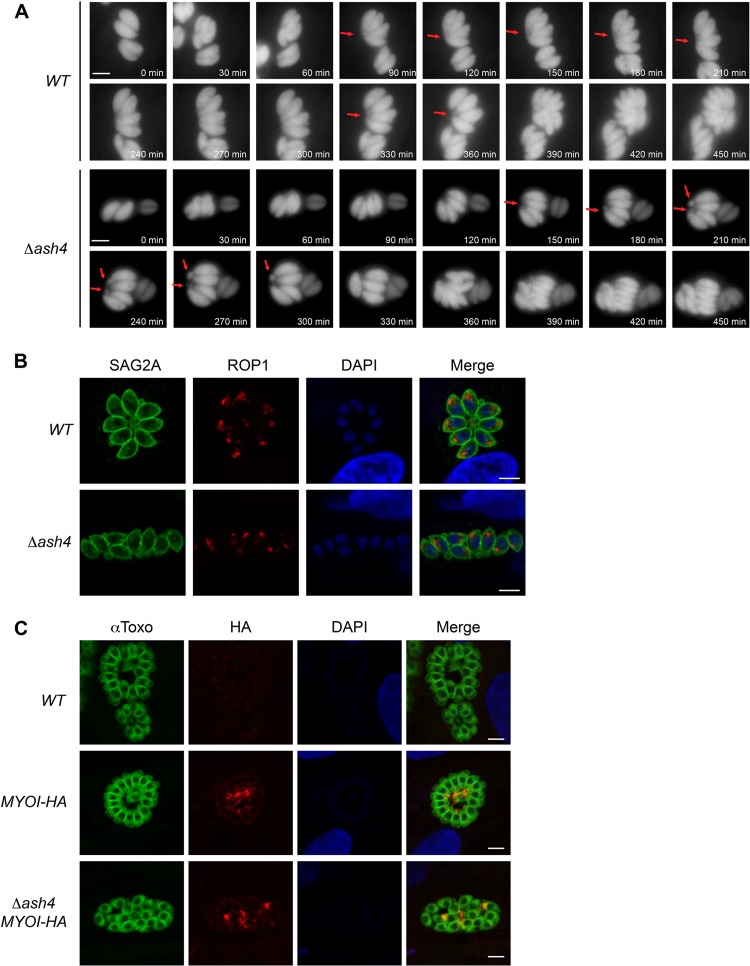
*Δash4* parasites form multiple residual bodies per vacuole. (A) Still frames from time-lapse microscopy of dividing wild-type and *Δash4* parasites. Red arrows indicate possible residual bodies. Time from the start of the movie is indicated in minutes. The scale bar is 5 μm. (B) Indirect immunofluorescence microscopy of ROP1 localization in wild-type and *Δash4* parasites. Anti-SAG2A signal is shown as a marker for parasites, anti-ROP1 highlights the apical end of the parasites, and DAPI shows nuclei. The scale bar is 5 μm. (C) Indirect immunofluorescence microscopy of MyoI-HA in untagged wild-type (*WT*), *MYOI-HA*, and *Δash4* (*Δash4 MYOI-HA*) parasites. “αToxo” indicates parasites, anti-HA stain indicates MyoI-HA localization, and DAPI highlights nuclei. The scale bar is 5 μm.

We hypothesized that failure of *Δash4* parasites to form rosettes was due to potential defects in cell division. Time-lapse microscopy of cell division showed that wild-type parasites stayed tightly associated, reoriented after each division, and maintained an ordered intravacuolar architecture (Fig. 4A; see also Movie S1 in the supplemental material). Frequently, we observed wild-type parasites forming a shared structure at their basal ends, likely the residual body (Fig. 4A; see also Movie S1). In contrast, the *Δash4* parasites appeared to be organized in pairs during division and failed to reorient after division, leading to the formation of disordered vacuoles (Fig. 4A; see also Movie S2). Additionally, the *Δash4* “pairs” frequently formed structures at their basal ends, suggesting that the knockout parasites form multiple residual bodies per vacuole (Fig. 4A; see also Movie S2). To determine if the *Δash4* parasites were arranged in pairs as the microscopy suggested, we performed immunofluorescence staining of the apical rhoptry protein 1 (ROP1) in wild-type and knockout strains. Consistent with the time-lapse imaging, mutant parasites were often found in close proximity to a second parasite oriented in the same direction (Fig. 4B). Recent work has demonstrated that myosin I (MyoI) is localized to the residual body (9). Consistent with our previous observations, we found that *Δash4* parasites formed multiple MyoI foci inside each vacuole, in contrast to the wild-type parasites, which formed a single MyoI focus at the center of each rosette (Fig. 4C; see also Fig. S4) as previously described (9).

10.1128/mSphere.00393-18.5 MOVIE S1 Time-lapse microscopy of wild-type parasites dividing. Parasites express a cytosolic Tandem tomato. Images were taken in 10-min increments. Video data represent 7 frames/s. The scale bar indicates 5 μm. The arrow on the first frame indicates the parasites followed in still frames in Fig. 4A. Download Movie S1, AVI file, 2.2 MB. Copyright © 2018 Foe et al. 2018 Foe et al. This content is distributed under the terms of the Creative Commons Attribution 4.0 International license.

10.1128/mSphere.00393-18.6 MOVIE S2 Time-lapse microscopy of *Δash4* parasites dividing. Parasites express a cytosolic Tandem tomato. Images were taken in 10-min increments. Video data represent 7 frames/s. The scale bar indicates 5 μm. The arrow on the first frame indicates the parasites followed in still frames in Fig. 4A. Download Movie S2, AVI file, 2.2 MB. Copyright © 2018 Foe et al. 2018 Foe et al. This content is distributed under the terms of the Creative Commons Attribution 4.0 International license.

10.1128/mSphere.00393-18.4 FIG S4 Generation of *MYOI-HA* and *Δash4 MYOI-HA* parasites. The cartoon depicts the tagging strategy. The red arrow indicates the approximate location of the CRISPR/Cas9 cut site. Black arrows indicate locations of *MYOI*-HA check primers. PCR on the untagged endogenous locus resulted in a 0.45-kb band; successful integration of tagging construct resulted in a band of approximately 2.7 kb. Data represent results of PCR performed with MYO-HA check primers in wild-type (*WT*), *Δash4*, *MYOI-HA*, and *Δash4 MYOI-HA* strains. Download FIG S4, PDF file, 0.2 MB. Copyright © 2018 Foe et al. 2018 Foe et al. This content is distributed under the terms of the Creative Commons Attribution 4.0 International license.

**FIG 5:**
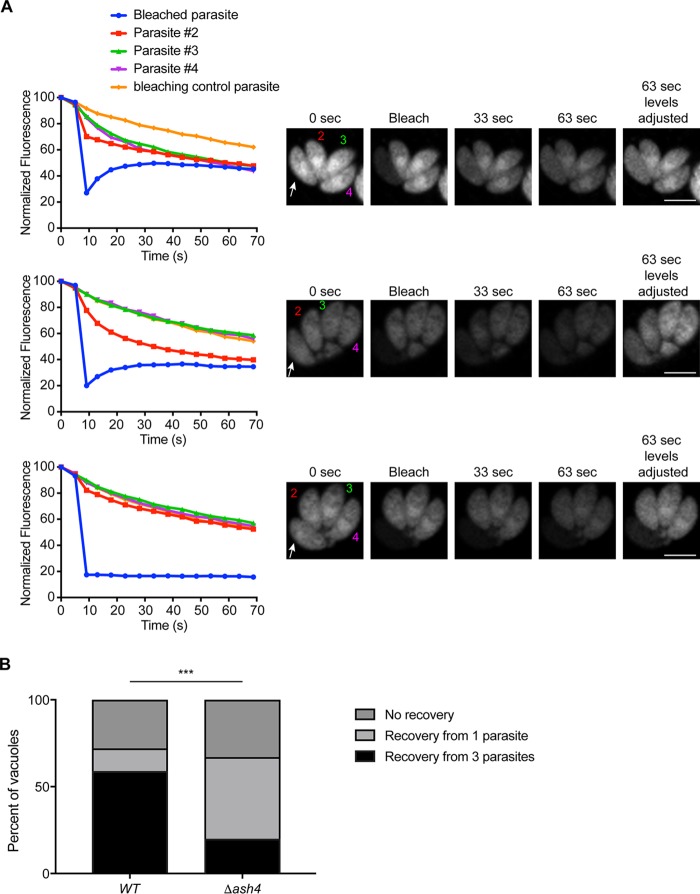
The majority of *Δash4* parasites are ordered in pairs. (A) Fluorescence recovery after photobleaching (FRAP) assays for wild-type and *Δash4* parasites. The top graph is representative of the data that result when a bleached parasite (white arrow; blue line in graph) recovers from all of the parasites in a vacuole (numbered parasites; distributed recovery). The middle graph shows data that are representative of recovery from a single parasite (single recovery). The bottom graph is representative of a parasite with no recovery. Graphs show the normalized fluorescence of each parasite in the vacuole, with an orange line depicting the normalized fluorescence of a parasite in a separate vacuole to indicate photobleaching due to imaging. Images at the right are representative of the results seen in the graphs and at various time points after bleaching of a single parasite as indicated. (B) Graph of the percentage of parasites that recovered by each of the three recovery types described in the panel A legend for wild-type and *Δash4* parasites. At least 30 vacuoles were counted for each strain. Statistical significance was calculated by the chi-square test using raw count numbers. ***, *P* < 0.001.

The residual body organizes the dividing parasites in the vacuole and allows diffusion of cytosolic proteins and small molecules between parasites (8–10). On the basis of the observations that *Δash4* parasites formed multiple residual bodies per vacuole and were oriented in a manner that suggests that they existed as pairs, we tested if each “pair” was connected via its own residual body. Since residual bodies mediate the transfer of cytosolic proteins between parasites, we performed fluorescence recovery after photobleaching (FRAP) on wild-type and mutant parasites expressing a diffusible cytosolic Tandem Tomato fluorophore to directly test parasite connectivity. We observed three strain-independent outcomes from the FRAP assays. First, we observed vacuoles in which the bleached parasite recovered fluorescence from all other parasites in the vacuole (distributed recovery), as previously reported (9, 10). Secondly, we observed vacuoles in which the bleached parasite recovered from a single parasite (single recovery). Finally, we observed vacuoles in which the bleached parasite failed to recover from any parasite (no recovery; Fig. 5A). Quantification of recovery outcomes for each strain revealed that nearly 60% of wild-type vacuoles had distributed recovery, in contrast to the *Δash4* vacuoles, where only 20% recovered in this manner (Fig. 5B). In contrast, we observed that only 10% of wild-type parasites but greater than 50% of *Δash4* parasites underwent single recovery (Fig. 5B). Regardless of the strain, nearly 30% of the vacuoles failed to recover at all (Fig. 5B). These data confirm that the majority of *Δash4* parasites were organized as true pairs of parasites connected via individual residual bodies.

**FIG 6:**
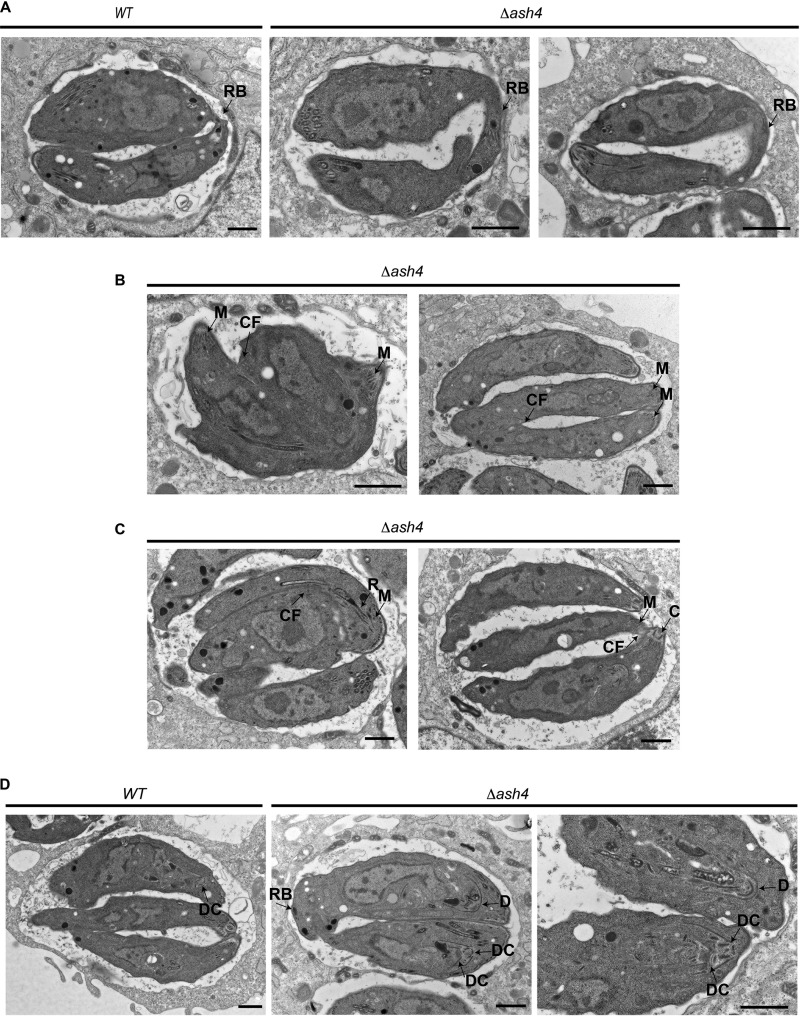
Loss of *ASH4* results in endodyogeny defects. (A) Electron micrographs of wild-type and *Δash4* parasites. Residual bodies are indicated by the arrow labeled RB. The scale bar is 1 μm. (B) Electron micrographs of *Δash4* parasites that have failed to complete division. The parasite apical end is indicated by an arrow highlighting micronemes (M). The arrow labeled “CF” indicates cleavage furrow. The scale bar is 1 μm. (C) Electron micrographs of *Δash4* parasites that have initiated division from their basal end. The apical end of parasites is indicated by arrows identifying micronemes (M), rhoptories (R), and the conoid (C). The unlabeled arrow indicates cleavage furrow. The scale bar is 1 μm. (D) Electron micrographs of wild-type and *Δash4* parasites forming daughter parasites. The arrow labeled “DC” indicates a daughter parasite conoid. The arrow labeled “D” indicates a developing daughter parasite. The scale bar is 1 μm.

**FIG 7:**
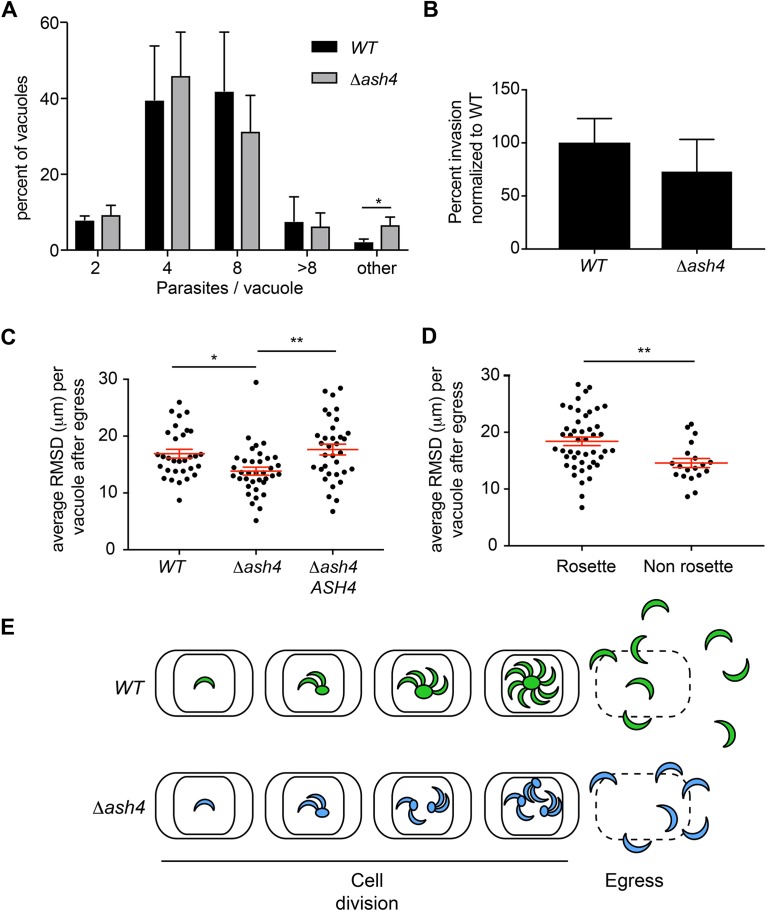
*Δash4* parasites have dispersion defects following egress. (A) Graph of average percentages of vacuoles that contain 2, 4, 8, >8, or “other” parasites (i.e., vacuoles with a non-power of 2 number of parasites or vacuoles with <8 parasites that could not be confidently counted). Error bars indicate standard deviations from the means. Results represent three independent experimental replicates with at least 80 vacuoles counted per experiment. Statistical significance was determined initially by chi-square analysis of counts, and data were significant (*P* < 0.05). To identify where the statistical significance lay, unpaired 2-tailed student’s T tests were performed. All statistically significant comparisons are shown (*, *P* < 0.05). (B). Graph of the average percentages of invasion of wild-type and *Δash4* parasites normalized to average percentage of wild-type invasion. Error bars represent standard deviations from the means. No statistical significance was found by an unpaired 2-tailed Student's *t* test. (C) Scatter plot of average root mean squared displacement (RMSD) of wild-type, *Δash4*, and ASH4 rescue (*Δash4 ASH4*) vacuoles at 50 s postegress. At least 30 vacuoles with 8 parasites were analyzed for each strain. Red lines indicate means ± standard errors of the means (SEM). One-way ANOVA was performed with a subsequent Tukey’s multiple-comparison test to determine significance, and all statistically significant comparisons are indicated (*, *P* <0.05; **, *P* < 0.01. (D) Scatter plot of average RMSD for wild-type and ASH4 rescue parasites based on whether they were derived from ordered rosettes or nonrosetted vacuoles at 50 s postegress. Red lines indicate means ± SEM. In total, 46 ordered vacuoles and 19 disordered vacuoles were used in the analysis. Statistical significance was determined by an unpaired 2-tailed Student's *t* test. **, *P* < 0.01. (E) Model for why *Δash4* parasites form small plaques. Wild-type parasites (green) successfully divide and have an ordered architecture and normal dispersion after egress. *Δash4* parasites (blue) have endodyogeny defects leading to fewer viable parasites overall and to parasites that organize as pairs, resulting in a disordered intravacuolar architecture, which leads to a reduction in the ability to efficiently disperse from host cells following egress.

We next used transmission electron microscopy (TEM) to examine how the loss of ASH4 altered the structure of the residual body. This analysis demonstrated that wild-type parasites formed short, thin residual bodies that were contiguous with both parasites, as previously reported (6, 8–10). In contrast, *Δash4* parasites formed wide, distended residual bodies that appeared largely derived from a single parasite (Fig. 6A). The electron micrographs also revealed multiple cell division defects in *Δash4* parasites. These defects included parasites that initiated cytokinesis from their apical end but that were unable to complete division (Fig. 6B) and parasites that incorrectly initiated division from their basal end (Fig. 6C). Most surprisingly, we observed knockout parasites that formed daughter parasites inside the second daughter parasite (Fig. 6D) instead of side by side as normally occurs during endodyogeny. These data suggest that, in addition to being organized as pairs, *Δash4* parasites also had defects in endodyogeny and cytokinesis. The centrosome is thought to control initiation of the formation of daughter parasites (7). To determine if *ASH4* is involved in the regulation of the centrosome, we performed immunofluorescence microscopy to examine Centrin1 in the *Δash4* and wild-type parasites. Surprisingly, we found no obvious defects in the localization or duplication of the centrosome in our mutant parasites (data not shown). On the basis of the TEM phenotypes, we performed parasites per vacuole assays to assess the rate of cell division in the wild-type and knockout parasites. These assays indicated no significant difference in the rate of *Δash4* parasite division compared to the rate seen with the wild-type parasites (Fig. 7A). However, consistent with the TEM images, there was a statistically significant (∼3-fold) increase in the number of *Δash4* vacuoles with an unusual number of parasites, consistent with a small but significant defect in parasite replication. To further verify if our ASH4 knockout parasites had endodyogeny defects, we searched for parasites that failed to complete division in our time-lapse video microscopy. Consistent with the TEM results and the assays analyzing the number of parasites per vacuole, we observed that a small fraction of *Δash4* parasites initiated but failed to complete cell division (Movie S3). However, based on the low frequency at which the *Δash4* parasites failed to divide, it seemed unlikely that the endodyogeny defect was the sole contributor to the plaque size defect.

10.1128/mSphere.00393-18.7 MOVIE S3 Time-lapse microscopy of *Δash4* parasites with division defects. Parasites express a cytosolic Tandem tomato. Images were taken in 10-min increments. Video data represent 7 frames/s. The scale bar indicates 5 μm. The arrow on the first frame indicates the parasites followed in still frames in Fig. 4A. Download Movie S3, AVI file, 2.5 MB. Copyright © 2018 Foe et al. 2018 Foe et al. This content is distributed under the terms of the Creative Commons Attribution 4.0 International license.

### Loss of *ASH4* leads to defects in radial dispersion from host cells after egress.

Plaque assays are sensitive to perturbations in multiple aspects of parasite biology, including cell division, host cell invasion, egress, and parasite dispersion. To identify additional factors that contributed to the small-plaque phenotype, we first determined if the mutant parasites had defects in host cell invasion. These assays conclusively demonstrated that *Δash4* parasites invade cells as well as wild-type parasites (Fig. 7B) and that the plaque phenotype was not invasion related. Previous work has shown that *Δgra2* parasites (which fail to rosette) are slow to externalize from host cells (8). On the basis of this observation, we sought to determine if our *ash4* mutant parasites also had defects in dispersion from host cells. We performed induced egress assays in which we used time-lapse microscopy to track the positions of individual egressed parasites over time. We found that at 50 s postegress, wild-type parasites, on average, spread radially 17 μm from their initial positions. In contrast, knockout parasites spread on average only 14 μm or 80% of the distance seen with the wild-type parasites (Fig. 7C). Importantly, the dispersion defect could be rescued by exogenous expression of *ASH4* in the knockout (*Δash4 ASH4*) parasites. This dispersion defect could be a direct consequence of losing ASH4 activity; however, it is possible that the inefficient dispersion was a consequence of the disordered intravacuolar architecture in the knockout parasites. To determine which factor (loss of ASH4 or lack of rosetting) contributed to the dispersion defect, we measured the distances that wild-type and *ASH4* rescue parasites travelled from either ordered or disordered vacuoles after egress. We found that the parasites that egressed from ordered vacuoles spread, on average, a radial distance of 18 μm in 50 s, while the parasites from disordered vacuoles spread only 83% of the average distance seen with the wild-type parasites (15 μm; Fig. 7D). These results suggest that the intravacuolar organization of parasites is important for their successful dispersion and may be important to maintain a robust infection.

## DISCUSSION

Serine hydrolases constitute a large class of enzymes that regulate many biological processes (14). Of particular interest is the T. gondii active serine hydrolase (ASH) family (13). The founding member of this family, TgPPT1, has been shown to possess depalmitoylase activity (12, 13), and it has been suggested on the basis of hidden Markov modeling and proteomic experiments that ASH2-4 may also function as depalmitoylases (13). The ASH4 enzyme is the only family member reported to be refractory to genetic disruption, making it potentially essential for parasite viability (13).

Previous studies suggest that ASH4 may function as a depalmitoylase (13). Data presented here demonstrate, however, that recombinant ASH4 (rASH4) does not process palmitoyl thioesters *in vitro* but instead processes short-acyl-chain esters and thioesters, suggesting that ASH4 may actually be a deacylase *in vivo*. However, we cannot rule out the possibility that additional factors *in vivo* may modulate the substrate specificity of ASH4. Factors that could influence ASH4 specificity include but are not limited to binding of protein cofactors and allosteric activation by small molecules. For instance, ASH4 is membrane localized, so it is possible that allosteric interactions with small molecules in the membrane and/or protein/protein interactions may modulate its substrate preference. However, while our *in vitro* data strongly suggest that ASH4 is not a depalmitoylase, a more extensive analysis of *in vivo* substrates will be essential to conclusively define the enzymatic function of ASH4. Such a study is likely to be challenging, as we currently know only what this enzyme is capable of processing *in vitro*, which may differ significantly from what it actually does process *in vivo*. Furthermore, without a clear idea of what its potential substrates may be and given the fact that other ASH family members may perform similar functions, it will likely be difficult to use genetic and proteomic/metabolomic methods to find native substrates *in vivo*.

Initial attempts to delete *ASH4* suggested that it was essential for parasite survival (13). We sought to conclusively determine if *ASH4* is essential and utilized a CRISPR/Cas9 system (16) to generate parasites lacking *ASH4*. This strategy was successful and demonstrated that while *ASH4* is not essential, it is important for parasite growth *in vitro*. Importantly, this work serves as a reminder that designating genes as essential for *in vitro* growth based solely on the inability to generate knockout lines is often problematic. Essentiality needs to be confirmed using other complementary approaches such as chemical inhibition or inducible knockdown/knockout of the target gene.

When parasites divide, they frequently arrange into organized patterns called rosettes that resemble petals of a flower around a central structure called a residual body (6, 8, 9). The biological function of the residual body and the consequence of rosette formation remain generally poorly understood. Recent work in multiple laboratories demonstrated the importance of connecting parasites to the residual body and showed that rosette formation is dependent on the residual body (8–10). Consistent with these findings, our data suggest that parasites that lack ASH4 fail to form rosettes as a consequence of premature scission from the residual body. Our data suggest a model where, when *Δash4* parasites divide, the sister parasites are initially tightly associated and are connected via a residual body, similarly to wild-type parasites. However, the *Δash4* parasites undergo premature scission from the residual body during parasite growth, resulting in two independent parasites in each vacuole. The two parasites then divide and form two functionally independent pairs of sister parasites, each with its own residual body. This cycle of division and scission is repeated and leads to the disordered intravacuolar architecture observed in the *Δash4* strain (Fig. 7E).

Previous work has shown that MyoI and MyoJ knockdown parasites are not connected via their residual bodies and that, as a consequence, they divide asynchronously inside the vacuole (9). We expected to observe a similar phenotype in our *ash4* knockout parasites based on the premature scission phenotype that we observed. However, the results of live video microscopy of the *Δash4* parasites suggested that our mutant divides synchronously for at least the first three divisions (when we can easily observe division). Why these early divisions are synchronous in our mutant is unknown. One plausible explanation is that, unlike the MyoI and MyoJ knockdown parasites, the *Δash4* parasites are frequently connected as pairs in the vacuole, and this connectivity may allow synchronous division in the early rounds of division. In later rounds of division, which we were not able to examine in our movies, the *Δash4* parasites may divide in an asynchronous manner.

The premature scission from the residual body observed in the *Δash4* parasites is consistent with a model where ASH4 plays a role in regulating/inhibiting cytokinesis. The cytosol of wild-type parasites in rosettes is contiguous, indicating that cytokinesis in dividing parasites is incomplete (8–10). If ASH4 functions as an inhibitor/regulator of cytokinesis as we propose, then loss of ASH4 could lead to the premature completion of cytokinesis, consistent with the premature scission phenotype that we observed. Additionally, we observed other cell division defects in the knockout parasites that are consistent with ASH4 regulating/inhibiting cytokinesis. By TEM, we observed parasites that initiated but failed to complete cytokinesis as well as parasites that incorrectly initiated cytokinesis from their basal end. Consistent with the TEM observations, we also observed at a low frequency *Δash4* parasites in the live video microscopy that initiated but failed to complete cytokinesis. While the cytokinesis hypothesis is attractive, to confirm it will require both a much greater understanding of ASH4 endogenous substrates and a greater understanding of cytokinesis in parasites.

The Mondragón laboratory has demonstrated that *gra2* knockout parasites, which fail to rosette, show a defect in the time required to externalize from host cells during egress (8). On the basis of the phenotypes seen in our experiments, we hypothesized that the loss of ASH4 might result in defects in the ability of parasites to move away from the site of egress. Consistent with this, we found that *Δash4* parasites have a defect in efficient dispersion following egress. While the data presented suggest that the loss of ASH4 directly causes the defect in dispersion, it is possible that the inefficient dispersion was due to disordered intravacuolar architecture (8). To test if the intravacuolar architecture can modulate dispersion, we compared the distances that the wild-type and *ASH4* rescue parasites traveled from ordered and disordered vacuoles. Surprisingly, we found that parasites from disordered vacuoles in which ASH4 was present also had a defect in dispersion in comparison to parasites from ordered vacuoles. These finding suggest that the intravacuolar architecture/arrangement of T. gondii has a functional consequence for parasites *in vitro*. Importantly, recent work has demonstrated that parasites infecting mice also share their cytosol due to connections via the residual body (9) and as a consequence likely adopt an ordered structure. Therefore, the dispersion defect that we observed in our *in vitro* studies may also play important roles in the successful establishment and maintenance of infections *in vivo*. Mechanistically, why disordered vacuoles result in defects in dispersion is unclear. One hypothesis consistent with our results is that parasites in ordered rosettes are oriented with their apical end facing the cytosol of the infected cells. This orientation provides a clear path for parasites to externalize and then disperse to other cells. Parasites from disordered vacuoles are oriented in multiple directions, an arrangement that forces them to navigate around each other during externalization and dispersion (Fig. 7E). This ultimately results in a shorter radial distance traveled over time and may affect the ability of parasites to both establish and maintain an infection.

## MATERIALS AND METHODS

### Parasite culture.

Parasites were grown in human foreskin fibroblasts (HFFs) using a mixture of Dulbecco’s modified Eagle’s medium (DMEM) with 10% FetalPlex animal serum complex (Gemini Biotech, catalog no. 100602), 2 mM l-glutamine, 100 μg/ml penicillin, and 100 μg/ml streptomycin. Parasites were cultured at 37°C in 5% CO_2_.

### Plasmid construction.

The 6×His-ASH4 bacterial expression construct (pIF22) was created by PCR amplification of the *ASH4* open reading frame (ORF) from the strain RH cDNA using primers GGCAGCCATATGGGGAACGCTCTGAAG and GAATTCGGATCCTCATCATCTGACGAGACGCG. The PCR product was digested with Nde1 and BamH1 and ligated into pET-28A. The catalytically dead allele (6×His-ASH4^S124A^; pIF22-S124A) was created by site-directed mutagenesis of pIF22 according to Stratagene’s QuikChange site-directed mutagenesis protocol using primers GTGTATGGCCGTgCCCTTGGCACCGGG and CCCGGTGCCAAGGGcACGGCCATACAC.

The ASH4^WT^-HA rescue construct (pIF52) was generated as detailed below. The endogenous 5′ untranslated region (5′UTR) of *ASH4* was PCR amplified from genomic DNA using primers GCGTAGTTAATTAACGAACTCCTCTGTAGCAGTAG and CCCCATGCTAGCCTCGAAAAACACCGAGAAGTTTC. The *ASH4* ORF with a single C-terminal HA tag was PCR amplified from pIF22 using primers TTCGAGGCTAGCATGGGGAACGCTCTGAAG and AGAGCTGCGGCCGCCTAAGCGTAATCTGGAACATCGTATGGGTATCTGACGAGACGCGC. The 3′UTR of *ASH4* was PCR amplified from genomic DNA using primers TACGCTTAGGCGGCCGCAGCTCTGCGTGCCATCAAAC and ATCGATAAGCTTTGCGAGACACACAAGGTGC. The 5′UTR was digested with PacI and Nhe1, the *ASH4* ORF was digested with Nhe1 and Not1, and the 3′UTR was digested with Not1 and HindIII. All three digested PCR products were ligated at the same time into uracil phosphoribosyl transferase (*UPRT*) integration vector (19) digested with Pac1 and HindIII.

The ASH4^G2A^-HA rescue construct (pIF55) was created using the Stratagene QuikChange site-directed mutagenesis protocol and plasmid pIF52 with the following primers: GAGGCTAGCATGGCGAACGCTCTGAAGCG and CGCTTCAGAGCGTTCGCCATGCTAGCCTC. The untagged *ASH4* rescue construct (pIF59) was created by PCR amplifying *ASH4* from pIF22 using primers TTCGAGGCTAGCATGGGGAACGCTCTGAAG and CAGAGCTGCGGCCGCTCATCATCTGACGAGACGC, and the PCR product was digested with Nhe1 and Not1 and ligated into pIF52.

The creation of the UPRT-Tandem Tomato integration plasmid (pIF56) was done by PCR amplification of the tubulin promoter driving Tandem Tomato from the pCRT2 plasmid (Boris Striepen laboratory) using primers ACTAGTGGATCCGCGGCTGGAG and CGGGTACCCGGGTTACTTGTACAGCTCGTCCATGC. This PCR product was digested with Xma1 and BamH1 and ligated into the *UPRT* integration vector.

The *Tg290860* integration plasmid (pIF58) was created by PCR amplifying the 5′UTR of *TgGT1_290860* from genomic DNA using ACGGAACCGGTGTTCCACGT and GGACCGGGCGCGCCAGCAGGCCACCCGAAAAAAAC. The 5′UTR was digested with Age1 and Asc1 and cloned into the UPRT integration vector using the same restriction sites. The 3′UTR of *TgGT1_290860* was amplified from genomic DNA by PCR using primers CGAGGGGGGGCCCAGAAACGCCACTGCGG and TTGCTCACATGTTCGACATCGATTTGAAATCCAACC. The 3′UTR was subsequently digested with Apa1 and PciI and ligated into the UPRT vector containing the 5′UTR for *Tg290860*. The Tg290860-Tandem Tomato plasmid (pIF60) was created by amplifying the tubulin promoter and Tandem Tomato ORF from pCRT2 with the following primers: CCTGCTGGCGCGCCCGGCTGGAGGCAACG and CGGGTACCCGGGTTACTTGTACAGCTCGTCCATGC. The PCR product was digested with Asc1 and Xma1 and ligated into the pIF58 plasmid.

CRISPR/Cas9 plasmids were generated from the *UPRT* targeting CRISPR/Cas9 plasmid designed by the Sibley laboratory (16). Guide RNA sequences for each gene were selected using the Eukaryotic Pathogen CRISPR guide RNA/DNA Design tool from the Tarleton group at the University of Georgia (20). Guide RNA sequences for each plasmid were changed using a Q5 mutagenesis kit (New England Biolabs, catalog no. E0554S). The reverse primer used for all Q5 reactions was CAACTTGACATCCCCATTTAC. Forward primers used to generate the specific gRNAs are listed below. The forward primer for the creation of the guide RNA for the deletion of *ASH4* (pIF81) was AGTGCGACGCGAGTTTCATCGTTTTAGAGCTAGAAATAGC. The forward primer for the generation of the guide used to C-terminally HA tag *MYOI* (pIF82) was CGTGAGCGAAGTCATGTAGAGTTTTAGAGCTAGAAATAGC. The primer used to generate the *Tg290860* integration gRNA CRISPR/Cas9 plasmid (pIF83) for integration at the *Tg290860* locus was GCGCTCTGGTACATCTGCTGGTTTTAGAGCTAGAAATAGC.

Plasmid pTKO2-Chlor was created by subcloning the chloramphenicol acetyl transferase from PH3CG_CAT_HA (Striepen laboratory) into pTKO2 plasmid (John Boothroyd laboratory), using standard molecular biology techniques.

### Strain construction.

All parasites used were RH parasites with a *Δku80* and *Δhxgprt* genetic background. The *ash4* deletion parasite strain (*Δash4*) was created as described below. The chloramphenicol acetyl transferase (CAT) gene from the pTKO2-Chlor plasmid was PCR amplified with primers containing 40 bases of homology to 5′ UTR and the 3′UTR of the endogenous *ASH4* gene. The primers used were TTTCTCTAGTCTACTGCGAAACTTCTCGGTGTTTTTCGAGTACTGGTGCTCGTATGCG and CGACGACCGCCCGCGAGGAAGTTTGATGGCACGCAGAGCTGCCTCGACTACGGCTTC. The underlined regions have homology to the 5′ and 3′ UTR of *ASH4*, respectively. Fifteen micrograms of the *ASH4* targeting PCR product and 3 μg of pIF81 were transfected into wild-type parasites. Selection for chloramphenicol resistance was done as previously described (21). Clonal lines were generated from the resistant population. Integration of the CAT into the *ASH4* locus was confirmed by PCR using the following primers: ACTTCCTAGCTGTAGGTCTAC and CGTCTTCTGACGGAAGAACC.

All *ASH4* rescue lines were created using a similar approach. Rescues were generated by transfecting 15 μg of a linearized (Psi1-digested) rescue plasmid (pIF52, pIF55, or pIF59) along with 3 μg of the CRISPR/Cas9 with the *UPRT* guide plasmid (16) into the *Δash4* parasites. Integration at *UPRT* locus was selected for using 5′-fluo-2′-deoxyuridine (FUDR) as previously described (19). Clonal parasite lines were generated from the FUDR-resistant parasites. PCR was used to demonstrate integration into the *UPRT* locus.

Tandem Tomato-expressing wild-type and *Δash4* parasites were created by integrating the pIF56 plasmid into the *UPRT* locus. Fifteen micrograms of linearized pIF56 was transfected along with 3 μg of the Sibley laboratory’s *UPRT*-targeting CRISPR/Cas9 plasmid (16). FUDR was used to select resistant parasites. Clonal lines were generated, and expression of Tandem Tomato was confirmed by microscopy. Tandem Tomato-expressing untagged *ASH4* rescue parasites were generated by transfecting 15 μg of linearized pIF60 and 3 μg of pIF83 into the untagged *ASH4* rescue strain (Δ*ash4 ASH4*). Integration was selected for using sinefungin as previously described (22). Clonal strains were isolated from the sinefungin-resistant parasites, and Tandem Tomato expression was verified by microscopy.

*MYOI-HA*-tagged parasites were generated as follows. An HA tag followed by the *GRA2* 3′UTR and *HXGPRT* ORF was PCR amplified from a previously described *CDPK1*-HA-tagging plasmid (23). The primers used (TGCGCGGCCGCGCACATTCGTCCAGTGGGGTACCCATATGACGTACCAGATTAC and CATTGCACTTTCCACACTCCGTGACAACCCGATCAGCACGAAACCTTGC) contained 30 bases of homology to the C terminus of *MYOI* and 30 bases of homology to the *MYOI* 3′UTR. Homology regions in primers are underlined. Fifteen micrograms of PCR product was transfected along with 3 μg of pIF82 into wild-type and *Δash4* parasites. Resistant parasites were selected with mycophenolic acid and hypoxanthine as previously described (24). Parasites were cloned, and the position of the tag was confirmed by PCR.

### Protein purification.

HIS6-ASH4 (rASH4) and HIS6-ASH4^S124A^ (rASH4^S124A^) were expressed from the pIF22 and pIF22-S124A plasmids in BL21-CodonPlus (DE3)-RIL Escherichia coli (Agilent Technologies, catalog no. 230245). Expression was induced with 0.25 mM IPTG (isopropyl-β-d-thiogalactopyranoside) for 4 h at 37°C. Recombinant proteins were purified as previously described (25) with the following modifications: 0.02% NP-40 detergent and 10% glycerol were included in both the lysis and wash buffer. Protein concentrations were quantitated by bicinchoninic acid (BCA) assay.

### *In vitro* biochemical assays.

4MU substrate assays were performed as previously described (25). Substrates were used at a final concentration of 10 μM. Enzymes were used at a 150 nM final concentration for the experiments represented in Fig. 1A. Enzymes were used at a final concentration of 50 nM for the experiments represented in Fig. S1B in the supplemental material. Fluorescence (λ_ex_ = 365 nm and λ_em_ = 455 nm) was measured every minute on a Cytation 3 imaging reader (BioTek, Winooski, VT, USA) for 60 min.

4-Nitrophenol acetate (4NPA) and 4-nitrophenol thiol-acetate (4-S-NPA) assays were performed as previously described for 4-nitrophenol octanoate (4NPO) (12). Enzymes were used at a final concentration of 150 nM. Absorbance was monitored on a Cytation 3 imaging reader (BioTek, Winooski, VT, USA).

QStE assays were performed as previously described. In short, a 10 μM final concentration of QstE substrate was incubated with 150 nM enzyme in reaction buffer (20 mM HEPES [pH 7.4], 150 mM NaCl, 10 mM CHAPS {3-[(3-cholamidopropyl)-dimethylammonio]-1-propanesulfonate}) at 37°C. Fluorescence (λ_ex_ = 410 nm and λ_em_ = 450 nm) was monitored every minute for 1 h on a Cytation 3 imaging reader (BioTek, Winooski, VT, USA).

### Plaque assays.

Parasites were isolated from host cells by syringe lysis and filtered with a 5-μm filter to remove host cell debris. The number of parasites per microliter was counted on a BD Accuri flow cytometer using forward and side scatter. A total of 200 parasites were added to confluent HFFs in 6-well dishes. Parasites were grown for 7 days, fixed with cold methanol, and stained with crystal violet. The plaque area was determined by manually measuring the area of each plaque using Image J.

### Live video microscopy.

Tandem Tomato-expressing parasites were incubated with confluent host cells grown on MatTek glass-bottomed microwell dishes (part no. P35G-1.5-14-C). At 6 to 8 h postinfection, parasites were imaged with epifluorescence on a Nikon Eclipse Ti microscope equipped with An iXon3 3888 electron-multiplying charge-coupled-device (EMCCD) camera (Andora) with a temperature- and humidity-controlled housing (37°C and 5% CO_2_). Images were taken every 10 min.

### Indirect immunofluorescence microscopy.

Parasites infected confluent HFFs on coverslips for 20 h, after which coverslips were fixed with cold methanol or, in the case of Fig. 4B, 4% paraformaldehyde. Cover slips were permeabilized with 0.1% Triton X-100 in 1× phosphate-buffered saline (PBS) and blocked in 3% bovine serum albumin (BSA) in 1× PBS for 30 min. For standard indirect immunofluorescence microscopy, the following antibodies were incubated overnight at 4°C in 3% BSA in 1× PBS at the specified dilution: anti-Toxo-fluorescein isothiocyanate (anti-Toxo-FITC) (Thermo Fisher, catalog no. PA1-7253) (1:1,000), anti-HA 3F10 (Roche, catalog no. 11867423001) (1:1,000), and anti-SAG2A and anti-ROP1 (John Boothroyd) (1:1,000). After incubation with primary antibodies, coverslips were washed 3 times with 1 ml 1× PBS. Alexa Fluor 488 and 594 antibodies (Life Technologies) were used as secondary antibodies and were incubated at 1:1,000 in 3% BSA in 1× PBS for 1 h, followed by three washes in 1× PBS. Mounting medium was used with DAPI (4′,6-diamidino-2-phenylindole; Vector Laboratories Inc., catalog no. H-1200) to mount coverslips to slides. Slides were imaged by confocal microscopy on an LSM 700 laser scanning confocal microscope. The intensity levels of the images were adjusted such that no data were removed from images.

Tyramide amplification was used to observe ASH4^WT^-HA and ASH4^G2A^-HA localization. Coverslips were setup, fixed, and blocked as described above. Primary antibodies were incubated overnight at 4°C in 3% BSA in 1× PBS. Primary antibodies were used at the following dilution: anti-HA 3F10 antibody at 1:1,000, Anti-SAG2A at 1:1,000, and anti-ROP2/3/4 at 1:1,000 (John Boothroyd). Secondary antibodies were incubated for 1 h at room temperature in 3% BSA in 1× PBS at the following dilutions: anti-rat IgG (H+L)-horseradish peroxidase (HRP) conjugate (Life Technologies, catalog no. A10549) at 1:200, anti-mouse Alexa Fluor 647 (Life Technologies catalog no. A21236) at 1:1,000, and anti-rabbit Alexa Fluor 594 (Life Technologies catalog no. A11012) at 1:1,000. After incubation with the secondary antibodies, tyramide amplification was performed using Tyramide signal amplification kit no. 2 (Life Technologies, catalog no. T20912). Mounting medium was used with DAPI (Vector Laboratories Inc., catalog no. H-1200) to mount coverslips to slides. Confocal imaging was done the same day with an LSM 700 laser scanning confocal microscope. Intensity levels were adjusted equally across all tyramide amplification images.

### Quantification of rosettes.

Parasites were grown in confluent HFF coverslips for 20 h. Coverslips were fixed and stained with the anti-Toxo-FITC antibody as described above. Slides were imaged on an LSM 700 laser scanning confocal microscope. Cover slips were counted blind, and at least 50 vacuoles with 8 parasites/vacuole were counted for each strain.

### Parasite lysates and Western blotting.

Parasites were lysed by incubation in lysis buffer (1× PBS, 0.5% NP-40, and.1% SDS) for 30 min on ice. Lysate was cleared by centrifugation at 14,000 rpm at 4°C. Samples were quantified by BCA assay. 3F10 anti-HA antibody was used at a 1:2,000 dilution, anti-SAG1 antibody (John Boothroyd) was used at a 1:10,000 dilution, AMA1 antibody UVT-59 (Gary Ward) was used at a 1:2,000 dilution, and the DJ1 antibody (Matt Bogyo) was used at a 1:2,000 dilution.

### Validation of myristoylation.

Parasites infecting HFFs were labeled with 25 μM alkynlated myristate (YnMyr; Tate Laboratory) (18) overnight. Labeled parasites were collected from host cells by syringe lysis. Parasites were lysed by incubation on ice with lysis buffer (1× PBS, 0.5% NP-40, 0.1% SDS) for 30 min, followed by centrifugation (14,000 rpm) at 4°C for 10 min. Protein concentrations were quantified by BCA assay. Two hundred micrograms of lysate was added to 30 μl monoclonal anti-HA-agarose (Sigma, catalog no. A2095), along with IP buffer (20 mM HEPES [pH 7.4], 150 mM NaCl, 0.5% NP-40, 0.1% SDS) to bring the reaction mixture to a 200-μl total volume. Immunoprecipitation and click reactions were carried out as previously described (26). HA Western blotting was done using the 3F10 HA antibody as described above.

### Fractionation assays.

Parasites were isolated from host cells by syringe lysis. Parasites were lysed by sonication in cold 1× PBS. Lysates were fractionated by ultracentrifugation at 100,000 × *g* for 60 min at 4°C. The soluble fraction was removed and quantified by BCA assay. The insoluble fraction was collected and solubilized by sonication in cold 1× PBS and quantified by BCA assay. Twenty-five micrograms of protein was loaded for each sample on standard SDS-PAGE gels. Western blotting was performed using antibodies as described above.

### Fluorescence recovery after photobleaching (FRAP).

Parasites expressing a cytosolic Tandem Tomato were allowed to infect confluent HFFs grown in MatTek microwell dishes for ∼16 h. FRAP assays were done with live parasites in a temperature-controlled chamber at 37°C on an LSM 700 microscope. The ZEN FRAP module was used with the following settings: the 555 laser was used at 30% power; vacuoles were imaged for 2 frames prebleach, followed by 6 iterations of bleaching; parasites were then imaged either every 4 or every 5 s for at least 1 min postbleaching. Vacuoles were not analyzed if a second parasite in the vacuole had a decrease of >30% in its initial fluorescence during bleaching. Intensity levels were adjusted to be equal across all images shown in this paper with the exceptions indicated.

### Invasion assays and assays of the number of parasites per vacuole.

Invasions assays were done with Tandem Tomato-expressing parasites as previously described (12). Parasites per vacuole assays were carried out as follows. Parasites were allowed to infect confluent HFFs on coverslips for 1 h, after which they were washed with warm media. At 20 h postinfection, coverslips were fixed and stained with anti-Toxo-FITC antibody as described above. Tile scans of 4-by-4 or 5-by-5 fields of view with z stacks were taken on a LSM 700 microscope. Each vacuole in the tile scan containing two or more parasites was counted; at least 80 vacuoles were counted for each sample. Vacuoles with unusual parasite numbers (not powers of 2) or vacuoles where the number of parasites was <8 but could not be confidently determined were counted as “other.” To confirm that parasites were in the same vacuoles, bright-field images were also taken.

### Transmission electron microscopy.

For ultrastructural analyses, infected cells were fixed in 2% paraformaldehyde (Electron Microscope Sciences, catalog no. 15710)–2.5% glutaraldehyde (Poly Scientific R&D Corp., catalog no. S1809-8oz)–100 mM sodium cacodylate buffer (Electron Microscopy Sciences, catalog no. 11654) (pH 7.2) for 1 h at room temperature. Samples were washed in sodium cacodylate buffer, embedded in a thin layer of 2.5% agarose, and postfixed in 1% osmium tetroxide (Polysciences Inc.) for 1 h. Samples were then rinsed extensively in double-distilled water (dH_2_O) prior to *en bloc* staining with 1% aqueous uranyl acetate (Ted Pella Inc., Redding, CA) for 1 h. Following several rinses in dH_2_O, samples were dehydrated in a graded ethanol series and embedded in Eponate 12 resin (Ted Pella Inc.). Sections of 95 nm were cut with a Leica Ultracut UCT ultramicrotome (Leica Microsystems Inc., Bannockburn, IL), stained with uranyl acetate and lead citrate, and viewed on a Jeol 1200 EX transmission electron microscope (Jeol USA Inc., Peabody, MA) equipped with an Advanced Microscopy Techniques (AMT) 8-megapixel digital camera and AMT Image Capture Engine V602 software (Advanced Microscopy Techniques, Woburn, MA).

### Induced-egress assays.

Assays were carried out as previously described (27) with minor changes. MatTek microwell dishes with subconfluent HFFs were infected with Tandem Tomato-expressing parasites for ∼20 h. Infected dishes were washed 2× with warm Hanks’ balanced salt solution (HBSS) and then incubated with warm egress buffer (HBSS, 1 mM MgCl_2_, 1 mM CaCl_2_, 10 mM NaHCO_3_, 20 mM HEPES [pH 7.1]). A23817 (Sigma catalog no. C7522-5MG) was added to the dish to achieve a final concentration of 5 μM. Real-time parasite egress was recorded at a frame rate of 2 frames/s on a Nikon Eclipse Ti microscope equipped with an iXon3 3888 EMCCD camera (Andora), with a 20× objective and a temperature- and humidity-controlled chamber at 37°C with 5% CO_2_. To quantify the spread of parasites during egress, we performed root means squared displacement (RMSD) analysis. In short, we measured the distance of each parasite to the mean parasite starting position from a single vacuole at 50 s postegress. The first time point where parasites began to move was assigned by the researcher, and the image frame at 50 s postegress was automatically selected for analysis. Using a custom MATLAB pipeline, each selected movie frame was segmented by assigning a threshold value corresponding to the fluorescence image, and the centroid position was calculated for each segmented parasite mask. The RMSD value was calculated for each vacuole according to the following equation: RMSD = [<(*x* − <*x*>)^2^> + <(*y* − <*y*>)^2^>]^1/2^.
