# Erratum for Foe et al., “The Toxoplasma gondii Active Serine Hydrolase 4 Regulates Parasite Division and Intravacuolar Parasite Architecture”

**DOI:** 10.1128/mSphere.00535-18

**Published:** 2018-10-10

**Authors:** Ian T. Foe, Ouma Onguka, Katherine Amberg-Johnson, Rikki M. Garner, Neri Amara, Wandy Beatty, Ellen Yeh, Matthew Bogyo

**Affiliations:** a Department of Pathology, Stanford University School of Medicine, Stanford, California, USA; b Microbiology and Immunology, Stanford University School of Medicine, Stanford, California, USA; c Biophysics Program, Stanford University School of Medicine, Stanford, California, USA; d Department of Molecular Microbiology, Washington University School of Medicine, Saint Louis, Missouri, USA; e Department of Biochemistry, Stanford University School of Medicine, Stanford, California, USA; f Chan Zuckerburg Biohub, San Francisco, California, USA

## ERRATUM

Volume 3, no. 5, e00393-18, 2018, https://doi.org/10.1128/mSphere.00393-18. The ToxoDB identifier for *ASH4* should be TgME49_262490 instead of TgME49_264290. The incorrect identifier appears twice in the PDF version of the article: line 5 of the abstract and line 26 of page 2.

