# Supplementary material for: The Toxoplasma gondii Active Serine Hydrolase 4 Regulates Parasite Division and Intravacuolar Parasite Architecture
Source: mSphere. 2018 Sep 19;3(5):e00393-18. doi: 10.1128/mSphere.00393-18 (PMC6147133; doi:10.1128/mSphere.00393-18)
Supplement: FIG S1 [file sph005182644sf1.pdf]

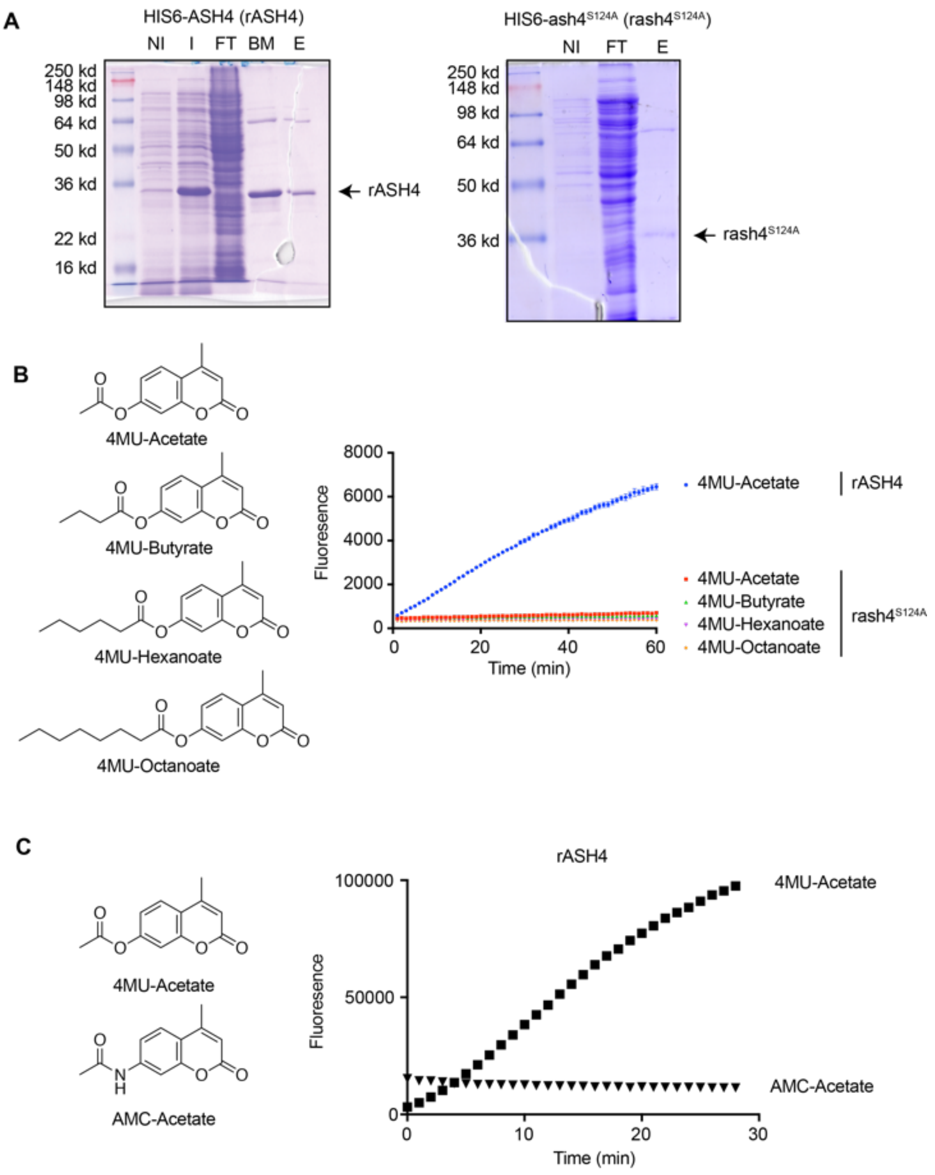

Supplemental Figure 1: Recombinant expression and activity of ASH4. **A) C**oomassie stained gel image (left) showing expression and purification of recombinant ASH4 (rASH4). Coomassie stained gel (right) showing expression and purification of recombinant catalytic dead ASH4 (rash4^{S124A}). NI indicates not induced sample; I indicates induction sample; FT indicates flow through; BM indicates bead bound material; E indicates elution from beads. Arrow indicates rASH4, and rash4^{S124A}. **B)** Structures of ester substrates and graph of a representative progress curve for rash4^{S124A} cleavage of the substrates. Error bars show the +/- SD. Cleavage of 4MU-Acetate by rASH4 shown as a positive control. Graph is the average of 3 technical replicates. **C)** Substrate structures and graph of a representative progress curve for rASH4 cleavage of 4-methyl-2-oxo-2*H*-chromen-7-yl acetate (4MU-Acetate), and *N*-(4-methyl-2-oxo-2*H*-chromen-7-yl) acetamide (AMC-Acetate) +/- SD. Graph is the average of three technical replicates.
