# Supplementary material for: The Toxoplasma gondii Active Serine Hydrolase 4 Regulates Parasite Division and Intravacuolar Parasite Architecture
Source: mSphere. 2018 Sep 19;3(5):e00393-18. doi: 10.1128/mSphere.00393-18 (PMC6147133; doi:10.1128/mSphere.00393-18)
Supplement: FIG S2 [file sph005182644sf2.pdf]

A

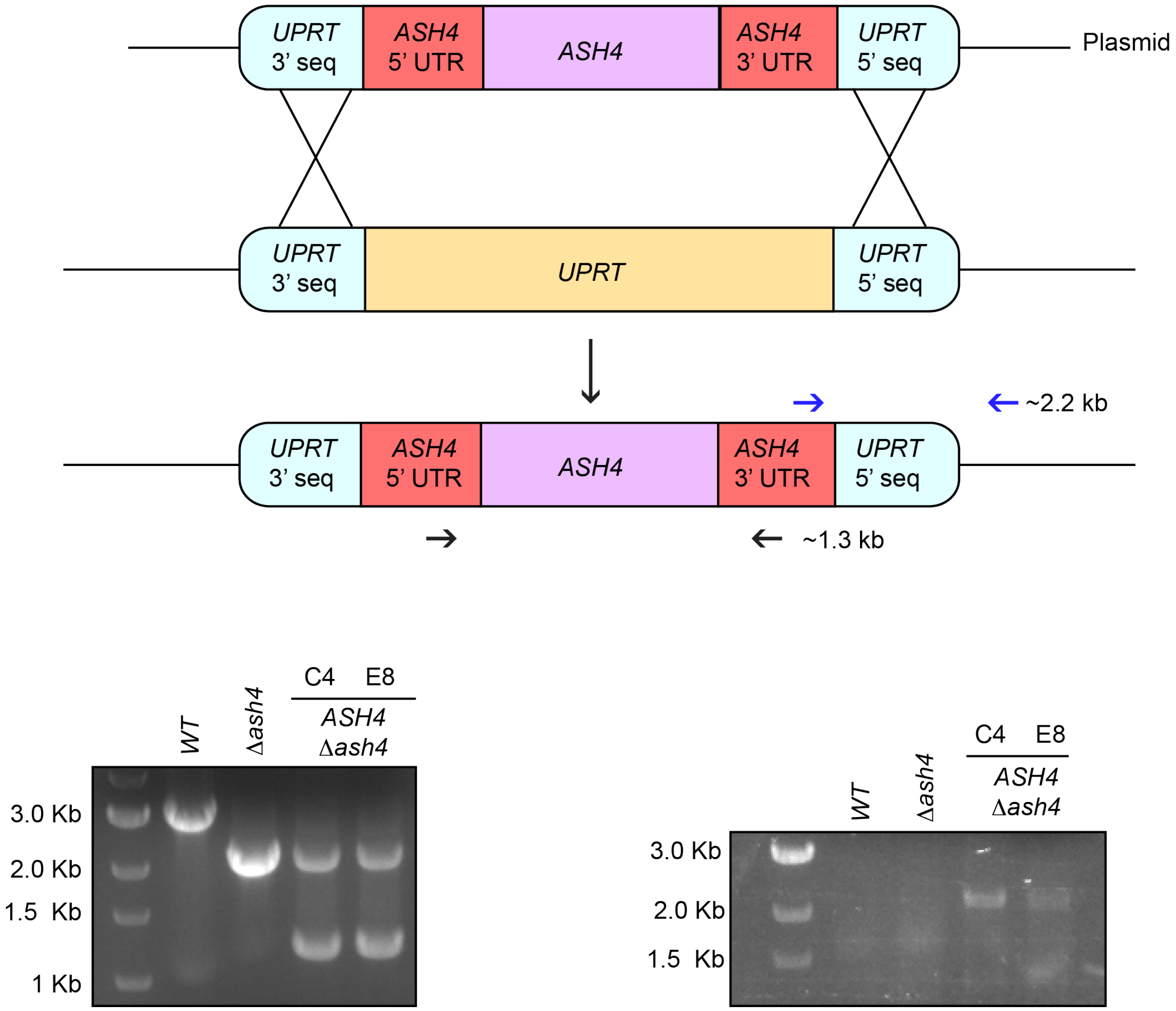

Supplemental Figure 2: Generation of the *ASH4* (*Δash4 ASH4*) rescue strain**.** Cartoon illustrating *ASH4* integration at the UPRT locus. Black arrows indicate primers for confirming the presence of ASH4. Primers lie both in the UTR regions of the rescue construct and the endogenous ASH4 locus. Primers test for integration of the rescue construct and the *Δash4* status. Blue arrows indicate location of UPRT integration check primers. Left gel shows PCR confirming integration of ASH4 rescue construct. PCR on ASH4 rescue strains generates both a 2kb band indicating *Δash4*, and a 1.3kb band corresponding to the rescue construct. Two clones are shown for ASH4 rescue, clone C4 was used for experiments in paper. Right gel shows successful integration of the rescue construct at the UPRT locus. Successfully integration results in a PCR product of 2.2 kb.
