# Supplementary material for: The Toxoplasma gondii Active Serine Hydrolase 4 Regulates Parasite Division and Intravacuolar Parasite Architecture
Source: mSphere. 2018 Sep 19;3(5):e00393-18. doi: 10.1128/mSphere.00393-18 (PMC6147133; doi:10.1128/mSphere.00393-18)
Supplement: FIG S3 [file sph005182644sf3.pdf]

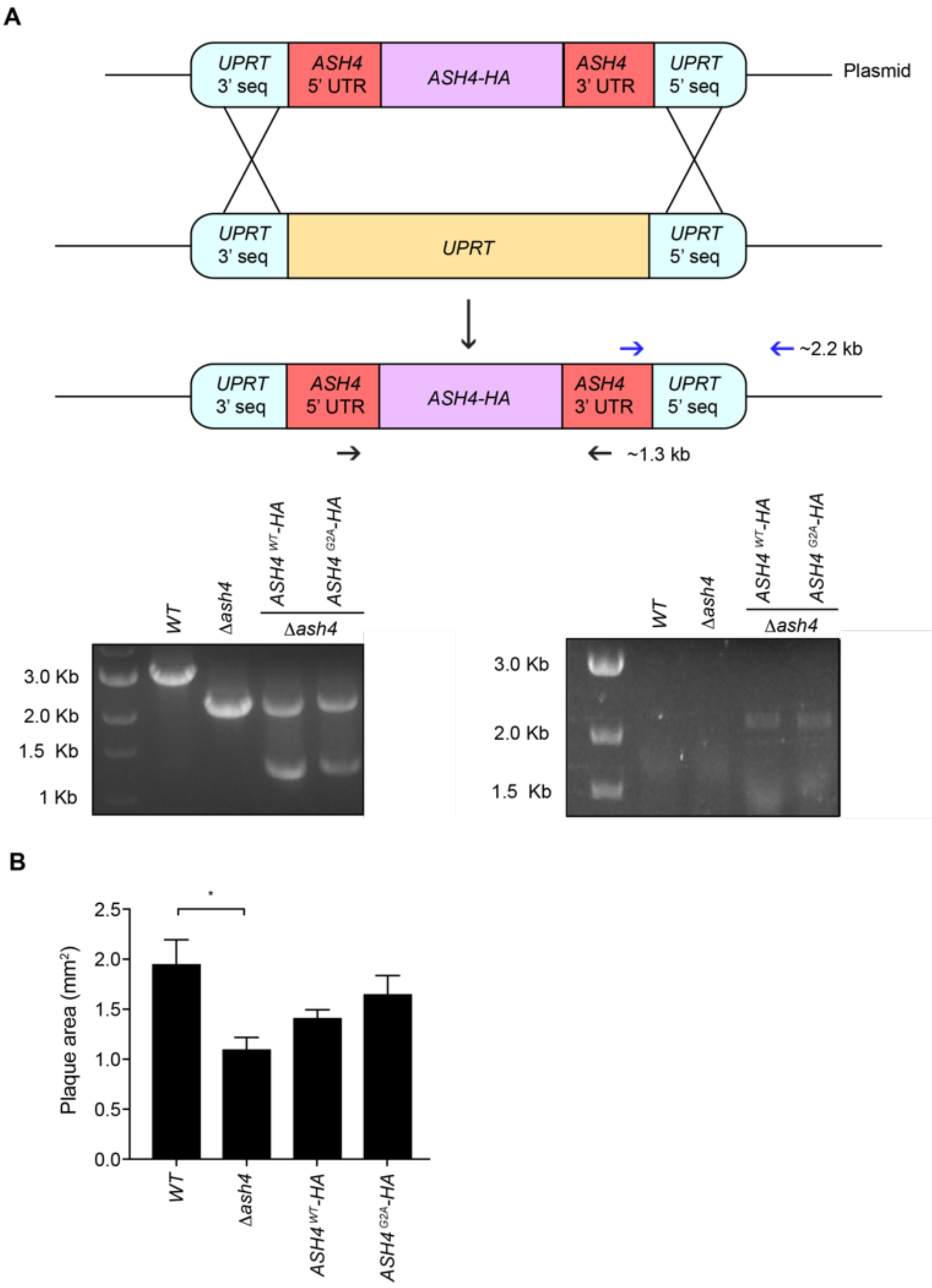

Supplemental Figure 3: Generation of *ASH4^{WT}-HA* and *ASH4^{G2A}-HA* rescue strains**. A)** Cartoon illustrating how the *ASH4-HA* alleles were introduced into the UPRT locus using strategy described in Supplemental Figure 2. Left gel shows PCR using primers to demonstrate integration of ASH4-HA rescue construct. Right gel shows PCR with primers for confirming construct integration into the UPRT locus. **B)** Graph depicts average plaque size of wild type (WT), *Δash4, ASH4^{WT}-HA* and *ASH4^{G2A}-HA* parasites in mm^2 +/- SEM. Experiment was performed 3 independent times in technical triplicate. One way ANOVA was performed followed by Tukey's multiple comparison test to determine statistical significance, all statistically significant comparisons indicated, * indicates p value < 0.05.
