# Supplementary material for: The Toxoplasma gondii Active Serine Hydrolase 4 Regulates Parasite Division and Intravacuolar Parasite Architecture
Source: mSphere. 2018 Sep 19;3(5):e00393-18. doi: 10.1128/mSphere.00393-18 (PMC6147133; doi:10.1128/mSphere.00393-18)
Supplement: FIG S4 [file sph005182644sf4.pdf]

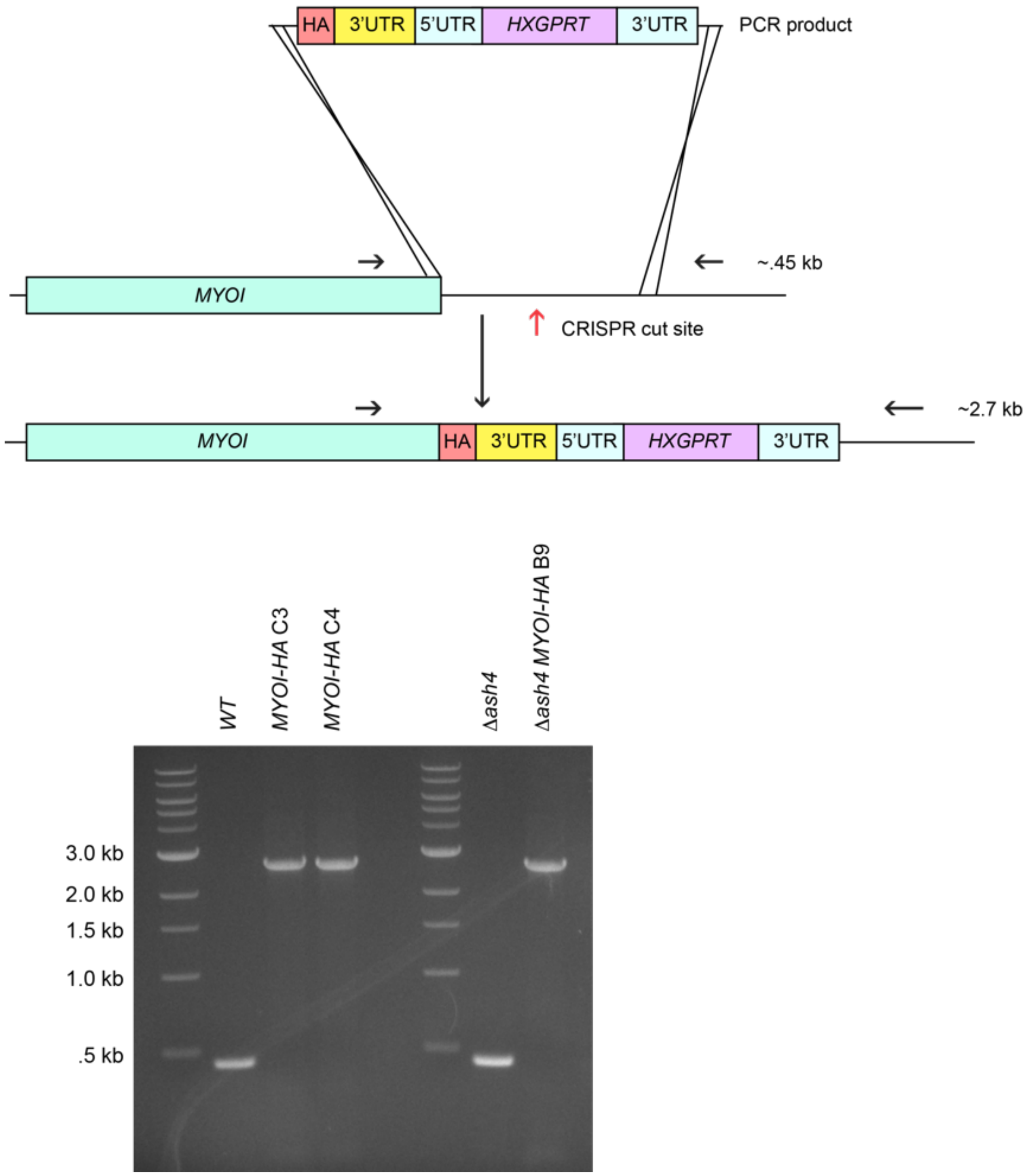

Supplemental Figure 4: Generation of *MYOI-HA*, and *Δash4 MYOI-HA* parasites. Cartoon depicting tagging strategy. Red arrow indicates approximate location of the CRISPR/Cas9 cut site. Black arrows indicate location of *MYOI*-HA check primers. PCR on the untagged endogenous locus results in a 0.45kb band, successful integration of tagging construct results in a band of approximately 2.7kb. PCR shown using MYO-HA check primers in wild type (*WT*), *Δash4, MYOI-HA* and *Δash4 MYOI-HA* strains.
